# Feijoa Fruit Peel: Micro-morphological Features, Evaluation of Phytochemical Profile, and Biological Properties of Its Essential Oil

**DOI:** 10.3390/antiox8080320

**Published:** 2019-08-19

**Authors:** Antonella Smeriglio, Marcella Denaro, Clara De Francesco, Laura Cornara, Davide Barreca, Ersilia Bellocco, Giovanna Ginestra, Giuseppina Mandalari, Domenico Trombetta

**Affiliations:** 1 Department of Chemical, Biological, Pharmaceutical and Environmental Sciences, University of Messina, Viale Palatucci, 98168 Messina, Italy; 2 Foundation Prof. Antonio Imbesi, University of Messina, P.zza Pugliatti 1, 98122 Messina, Italy; 3 Department of Earth, Environment and Life Sciences, University of Genoa, Corso Europa 26, 16132 Genoa, Italy

**Keywords:** *Acca sellowiana* (O. Berg) Burret, feijoa, essential oil, antioxidant, antimicrobial, cytoprotective

## Abstract

*Acca sellowiana* (O. Berg) Burret (Feijoa) is an evergreen shrub, belonging to the Mirtaceae family. The aim of this study was to investigate the micromorphological features of the feijoa fruit peel and to evaluate the phytochemical profile, as well as the antioxidant, cytoprotective, and antimicrobial properties of its essential oil (EO), by several in vitro cell-free and cell-based assays. The micromorphological analysis showed several schizogenic secretory cavities, immediately below the epidermal layer. Forty compounds were identified and quantified by GC-FID and GC-MS analyses. Sesquiterpenes were the most abundant ones (76.89%), followed by monoterpene hydrocarbons (3.26%), and oxygenated monoterpenes (0.34%). The main compounds were γ-Selinene (17.39%), α-Cariophyllene (16.74%), β-Cariophyllene (10.37%), and Germacene D (5.32%). The EO showed a strong and dose-dependent antioxidant, and free-radical scavenging activity. Furthermore, it showed cytoprotective activity on the lymphocytes, that have been pre-treated with 100 μM tert-butyl-hydroperoxide (t-BOOH), as well as a decrease in intracellular reactive oxygen species (ROS), induced by t-BOOH on erythrocytes. A preliminary antimicrobial screening against GRAM+ and GRAM− bacteria, as well as on fungi highlighted that EO showed the best activity against *S. aureus* and *C. albicans* (MIC 2.7 mg/mL). In light of these results, feijoa fruit EO could find various applications, especially in the food, nutraceutical, and pharmaceutical fields.

## 1. Introduction

*Acca sellowiana* (Berg) Burret, commonly known as feijoa, is an evergreen shrub, belonging to the Myrtaceae family with slow growth, originating from the highlands of South America. The most known varieties (apollo, coolidge, gemini, mammoth, moore, and triumph) differ in the time of maturation, size, and quality of the fruits. Feijoa fruits having a length of 4–8 cm, are oval, with a bright waxy green skin and a light, dense, granular, honey-colored, sweet and juicy pulp, with translucent seeds. It possess a peculiar floral aroma and a sweet-acidulous taste [1].

The peel flavor is much more resinous than the pulp, so that it is usually discarded. However, in some recipes, such as different types of chutney, it is often used because of its pleasant floral scent. The feijoa aroma is derived largely from volatile esters methyl benzoate, ethyl benzoate, and ethyl butanoate. Although, these esters have been found in other fruits, a unique feature of the feijoa aroma is the high percentage of methyl benzoate, which confer to it the peculiar aroma [2].

The fruit is also characterized, like apples, by a very high amount of pectin.

It is common consumed fresh or as a juice, but it is also used by the food industry for the production of several confectionery products, such as sorbet, ice cream, and so on [3].

The fruit contains volatile aromatic compounds, such as terpenes, tannins, steroidal saponins, and polyphenols. Furthermore, it is a good source of vitamin C, iodine, calcium, magnesium, and dietary fibres. Feijoa exerts digestive and laxative properties and, given its low calorie content, it is considered a valid diet adjuvant [2].

In Brazil and Uruguay, apart from some small crops, the fruits are not cultivated on a commercial scale, although some are harvested from wild or domestic plants. The commercial development of the feijoa occurred mainly outside these countries, starting from Europe [2], because of Edouard Andrè, a well-known French botanist and horticulturist, who imported the plant as ornamental fruit tree, common in domestic gardens and in warm-temperate regions. In this way, the seeds and plant have been widely distributed, leading to its cultivation and selection of crops in France, Israel, Italy, Russia, California, and New Zealand. The latter holds the greatest production.

In the past, greater importance was attributed to the medicinal properties of the plant than to the fresh fruit. In traditional medicine, the infusion of the plant leaves were used to treat dysentery and cholera, especially in children. Still today, in some countries, homeopathic pharmacies sell feijoa tea for this purpose. However, the most powerful infusion can be obtained from the feijoa fruit-dried peel [2].

Although the feijoa fruit has not been extensively investigated, several studies are already available, in particular, on the fruit extract or its juice, which report several interesting health effects, such as antibacterial, antifungal, analgesic, anti-inflammatory, anti-ulcer, anti-tumor, and osteoblastic activity [4,5,6,7,8,9,10,11,12,13]. Although, the polyphenol extracts of the feijoa fruit are the most investigated, Piscopo et al. [14] reported an interesting antioxidant and antimicrobial activity of a protein fraction from the feijoa fruit against several Gram-positive and Gram-negative standards, as well as clinically isolated bacterial strains. Moreover, these activities increased 10 fold, and 2–4 fold, respectively, after in vitro gastrointestinal digestion [14].

However, to date, no data are available about the biological properties of feijoa fruit peel essential oils (EO).

EOs are complex mixtures of plant secondary metabolites, with well-known antioxidants, cyto-protective, anti-inflammatory, allopathic, and antimicrobial properties [15,16].

In particular, EO antimicrobial activity has been validated by several studies, which often open new research perspectives. Among them, the quantitative activity-composition relationships classification models, based on machine learning algorithms, have been recently developed, in order to investigate the chemical components of the EOs that are mainly involved in the inhibition of *Pseudomonas aeruginosa* and *Staphylococcus spp.* biofilm production [17,18].

It has been observed, in fact, that often, the antibacterial activity of EOs is not directly related to their bacteriostatic/bactericidal activity, but to the phenotypic change in the bacterial strains [17].

In light of this, these screening tools could be very useful in selecting the most effective EOs for preventing infections, especially into healthcare field [17,18].

The aim of the present study was intended to first characterize the micromorphological features of the feijoa fruit peel, by optical and scanning electron microscopy (SEM). Subsequently, the phytochemical profile of the EO, isolated from the fresh fruit peel (*Acca sellowiana* (Berg) Burret var. coolidge) was characterized by GC-FID and GC-MS analysis. Moreover, different biological properties, such as antioxidant, cytoprotective, and antimicrobial properties, were evaluated by several in-vitro cell-free and cell-based assays.

## 2. Materials and Methods

### 2.1. Chemicals

Commercially available terpene analytical standards were purchased from Extrasynthese (Lyon, France). Dichloromethane was GC-grade, and was purchased from Merck (Darmstadt, Germany). C7-C40 n-alkanes standard mix solution, as well as other analytical grade chemicals and solvents were purchased from Sigma-Aldrich (Milan, Italy).

### 2.2. Plant Material and Isolation of Essential Oil

Feijoa (*Acca sellowiana* (Berg) Burret var. coolidge) fruits were harvested on 4 December 2018 by a local farmer in Lamezia Terme (Catanzaro, Italy), and immediately sent to the laboratory. The fresh fruits peel (250 g) was manually peeled off and the EO isolated by hydrodistillation, according to the current European Pharmacopoeia method. The EO was dried on Na_2_SO_4_ and stored in a dark-sealed vial with nitrogen headspace until analysis. For chemical characterization, EO was diluted in dichloromethane (10%, *v/v*), whereas for the evaluation of its biological activities, a stock solution (100 mg/mL) in DMSO was properly diluted in methanol.

### 2.3. Micromorphological Evaluation

For light microscopy (LM), small pieces of fruit peel were excised from a zone of maximum diameter, and hand cut by a razor blade. Cross sections were processed with 1% phloroglucinol in 20% hydrochloric acid for lignin staining. For scanning electron microscopy (SEM), small pieces of fruit peel (1–1.5 cm^2^) were fixed in FineFIX working solution (Milestone s.r.l., Bergamo, Italy) with 70% ethanol, and left for 24 h at 4 °C [19].

Subsequently, the tissue samples were dehydrated by increasing the concentrations of ethanol, followed by a critical point drying in carbon dioxide, using a CPD processor (K850 2M Strumenti S.r.l., Rome, Italy). Dried specimens were then mounted on aluminum stubs and sputter-coated with gold. Finally, specimens were observed by a Scanning Electron Microscope (SEM) Vega3 Tescan LMU (Tescan USA Inc., Warrendale, PA, USA) at an accelerating voltage of 20 kV.

### 2.4. GC-FID and GC-MS Analysis

Gas chromatographic (GC) analysis was performed on an Agilent gas chromatograph 7890A, equipped with a flame ionization detector (FID) and a mass spectrophotometer (MS) 5975C (Agilent Technologies Santa Clara, CA, USA). The elution was carried out by a HP-5MS capillary column (30 mm, 0.25 mm coated with 5% phenyl methyl silicone, 95% dimethylpolysiloxane, 0.25 μm film thickness) by using helium as carrier gas (1 mL/min) according to the method reported by Smeriglio et al. [16]. The injection of EO (1 μL, 10% CH_2_Cl_2_ *v/v*) was done in split mode (50:1) setting the injector and detector temperature at 250 °C, and 280 °C, respectively, for GC-FID analysis, and 250 °C, and 180 °C, respectively for GC-MS analysis. In the latter case, the ionization voltage was set at 70 eV, the electron multiplier at 900 V, and the ion source and transfer line temperature at 230 °C, and 260 °C, respectively. Mass spectra data were acquired in scan mode in the *m/z* range 45-450 amu. The compounds were identified based on their Kovats retention index (KI), relative to a standard mixture of n-alkanes, the values reported in the literature [15], and matching the mass spectra data with those of the MS library (NIST 08). Moreover, a comparison of MS fragmentation patterns with those reported in literature, and, whenever possible, co-injection with commercial available standards (purity ≥ 99%) were carried out. The percentages of compounds were determined from their peak areas in the GC-FID profiles.

### 2.5. Antioxidant and Free-Radical Scavenging Activity

The antioxidant and free-radical scavenging activity of feijoa EO was evaluated by several in vitro assays, based on different mechanisms and reaction environments. The results were expressed as an inhibition percentage (%) of the oxidative/radical activity, calculating the half-maximal inhibitory concentration (IC_50_) with the respective confident limits at 95%.

#### 2.5.1. Total Phenolic Compounds

The total phenols content was evaluated according to Smeriglio et al [20]. Briefly, 50 μL of EO solution (1.25–10 μg/mL) was added to 500 μL of Folin-Ciocalteu reagent and 450 μL of deionized water. After incubation for 3 min, 500 μL of sodium carbonate (10% *w/v*) was added. The samples were left in the dark at room temperature (RT) for 1 h, vortexing every 10 min, and the absorbance was recorded at 785 nm, using an UV-Vis spectrophotometer (Shimadzu UV-1601, Kyoto, Japan). 

#### 2.5.2. DPPH Assay 

The free radical scavenging activity of EO against DPPH radical was evaluated, according to Smeriglio et al. [21]. Freshly DPPH methanol solution (10^−4^ M), was mixed with 37.5 µL of EO solution (range 12.5–100 µg/mL) vortexing for 10 s. The decrease in absorbance at 517 nm, against a blank, was measured after 20 min by UV-Vis Spectrophotometer (Shimadzu UV-1601, Kyoto, Japan).

#### 2.5.3. Trolox Equivalent Antioxidant Capacity (TEAC) Assay 

The free-radical scavenging activity against ABTS radical was carried out, according to Smeriglio et al. [15]. The reagent solution (4.3 mM potassium sulfate and 1.7 mM ABTS Solution 1:5 *v/v*) was incubated, in the dark at RT, for at least 12 hours and used within 16 h, by dilution with phosphate buffer (pH 7.4) in order to reach an absorbance of 0.7 ± 0.02 at 734 nm. Fifty microliters of EO solution (1–8 µg/mL) was added into 1 mL of the reagent solution and incubated in the dark at RT for 6 min. An UV-VIS Spectrophotometer (Shimadzu UV-1601, Kyoto, Japan) recorded the absorbance at 734 nm.

#### 2.5.4. Ferric Reducing Antioxidant Power (FRAP) Assay

The assay was carried out according to Smeriglio et al. [20]. Freshly working FRAP reagent was warmed at 37 °C. Then, 50 µL of EO solution (1–8 µg/mL) were added into 1.5 mL of reagent, and the absorbance was measured after 4 min at 593 nm, by UV-VIS Spectrophotometer (Shimadzu UV-1601, Kyoto, Japan).

#### 2.5.5. Oxygen Radical Absorbance Capacity (ORAC) Assay 

The free radical scavenging activity against AAPH radical was tested, according to Bellocco et al. [22]. Briefly, 20 µL of EO solution (2–16 µg/mL) were dissolved in 75 mM phosphate buffer (pH 7.4) and pre-incubated for 15 min at 37 °C with 120 µL of fresh daily fluorescein solution (117 nM). After that, 60 µL of fresh daily 40 mM AAPH solution was added monitoring the reaction every 30 s for 90 min (λ_ex_: 485; λ_em_: 520) by a fluorescence plate reader (Fluostar Omega, BMG Labtech, Ortenberg, Germany).

#### 2.5.6. β-Carotene Bleaching Assay

β-Carotene bleaching assay was carried out according to Smeriglio et al. [23] by mixing fresh daily β-carotene emulsion with EO solution (5–40 μg/mL). A blank emulsion (without β-carotene) was used as negative control. The absorbance was recorded at the starting time (T = 0) at 470 nm and then monitored every 20 min, incubating the sample solution at 50°C in a water bath. The reaction was stopped at 120 min. Butylated hydroxytoluene (BHT) 1 mg/mL was used as a reference compound. The results were expressed as kinetic curves, and showed the capacity for Feijoa EO to counteract the heat-induced β-carotene bleaching.

#### 2.5.7. Iron-Chelating Activity

The iron-chelating activity of EO was evaluated, according to Smeriglio et al. [20]. Briefly, 50 μL of FeCl_2_∙4H_2_O solution (1.8 mM) was added to 100 μL of EO solution (1–8 μg/mL) and incubated at RT for 5 min. After that, 100 μL of ferrozine solution (4 mM) was added to the reaction mixture, that was diluted to 3 mL with deionized water, mixed and incubated for 10 min at RT. The absorbance was read at 562 nm, using a UV-VIS spectrophotometer (Shimadzu UV-1601, Kyoto, Japan). The results were expressed as inhibition (%) of the of Fe^2+^ chelating capacity, by calculating the half-maximal inhibitory concentration (IC_50_), with the respective confident limits at 95%.

### 2.6. Cell-based Assays

#### 2.6.1. Lymphocyte Isolation

The isolation of lymphocytes was performed according to Barreca et al. [24], with few changes. Heparinized whole blood from healthy volunteers (who have provided their written medical histories by a standardized questionnaire) has been a starting point for the lymphocytes isolation. The samples were diluted with equal volumes of PBS, layered over Histopaque-1077 in centrifuge tubes, and underwent centrifugation at 400 g for 30–40 min at 25 °C. The obtained cells was collected with a pipette, washed by centrifugation, and diluted with PBS. The peripheral blood mononuclear cells (PBMCs) were separated through a Percoll gradient, according to Repnik et al. [25]. They were then counted with a haemocytometer and suspended in Roswell Park Memorial Institute (RPMI) 1640 medium, added with 2 mM glutamine, 10% fetal calf serum, 100 units/ml streptomycin and penicillin G.

#### 2.6.2. Cytotoxicity and Cytoprotective Assays

The cytotoxicity assay was performed on cells (1 × 10^5^ /mL) incubated in complete medium with, or without, 150, 125, 100, 75, 50, 25, 10, 5, 2.5 and 1.25 μg/mL of feijoia EO for 24 h. The cyto-protective assay were performed on cells (1 × 10^5^ /mL) incubated in complete medium with, or without, 40, 20, 10, 5, 2.5, and 1.25 μg/mL of feijoia EO for 24 h, in the presence of 100 μM tert-butyl hydroperoxide (t-BOOH). The stock solution of feijoia EO was conveniently diluted with PBS to obtain, in the final reaction mixture, a concentration of the solvent below 0.1%. 

Trypan blue staining was utilized to test the viability of the completed incubation time, utilizing a haemocytometer and diluting an aliquot of the cell suspension (1:1, *v/v*) with 0.4% trypan blue.

Cytotoxicity has been also tested by lactate dehydrogenase (LDH) release into a culture medium from damaged cells. A commercially available kit from BioSystems S.A was utilized to analyze LDH activity in the medium, where the total amounts of the enzyme are present in the cells, after lysis by sonication [22,26]. Feijoia EO, at the final concentrations used, did not show any interference with the performed tests.

#### 2.6.3. Red Blood Isolation

The isolation of erythrocytes was performed according to Barreca et al. [27] with few changes. Heparinized whole blood from healthy volunteers (who have provided written medical histories by a standardized questionnaire) has been the starting point for the red blood cells isolation. Erythrocytes were separated by centrifugation (2500 rpm for 5 min) from the plasma and buffy coat, then washed three times with 10 volumes of 0.9% NaCl. Finally, the packed cells were diluted in 10 volumes of PBS and utilized for the following experiments.

#### 2.6.4. Assay for Erythrocyte Hemolysis

The erythrocyte hemolysis assay was carried out according to Barreca et al. [27]. Different amount of feijoia EO (150, 125, 100, 75, 50, 25, 10, 5, 2.5 and 1.25 μg/mL) were joined in a final volume of 1.0 mL, with erythrocytes (10% in PBS, pH = 7.4). The reaction mixes were incubated for 2 hours at 37 °C in a water bath, diluted with PBS (1:9, *v/v*), and centrifuged. The complete haemolysis in the reference sample was achieved by adding 8 volumes of distilled water centrifuging. Another sample without additives was diluted with the same volume of PBS, rather than distilled water, in order to eliminate any interference of spontaneous haemolysis. The absorbance of supernatant, after centrifugation, was analyzed at 540 nm and expressed as a percentage of complete hemolysis.

#### 2.6.5. Quantification of Intracellular Reactive Oxygen Species (ROS)

The ROS quantification was carried out according to Peter et al. [28]. The compound 2’,7’-dichlorodihydrofluorescein diacetate has been utilized as a dye, in order to detect the amount of intracellular ROS. The cells were incubated with t-BOOH (10 mM) for 2 h in presence/absence of different concentration of feijoa EO (40, 20, 10, 5, 2.5 and 1.25 μg/ml final concentration). The EO was added to the culture medium 30 min before the t-BOOH treatment. Then, the cells were washed twice with PBS (pH = 7.4) and incubated with 2’,7’-dichlorodihydrofluorescein diacetate for 30 min at 37 °C. The incubation time was completed and the fluorescence data were acquired at 525 nm following excitation at 488 nm. The ROS formation has been expressed as the maximum amount of radicals obtained in the samples treated with t-BOOH.

### 2.7. Antimicrobial Activity

The following strains were obtained from an in-house culture collection (University of Messina, Messina, Italy): *Staphylococcus aureus* ATCC 6538P, *S. aureus* ATCC 43300, three clinical strains of *S. aureus* obtained from the pharynges (strains 526, 530, 808), two clinical strains of *S. aureus* obtained from duodenal ulcers (strains 8, 14), two clinical strains of *S. aureus* obtained from hip prostheses (strains 6, 84); *Staphylococcus epidermidis* ATCC 35984; *Pseudomonas aeruginosa* ATCC 9027; *Escherichia coli* ATCC 10536; *Candida albicans* ATCC 10531, three clinical strains of *Candida albicans*, two clinical strains of *Candida glabrata*, two clinical strains of *Candida parapsilosis*. *S. aureus* strains, were recently characterized in terms of lipid profiles, and their correlation with antibiotic resistance and hydrophobicity [29]. *Candida sp.* clinical strains were identified by species-specific PCR-based methods [30].

All bacterial strains were cultured in Muller Hinton Broth (MHB, Oxoid, CM0405) at 37 °C (24 h). Candida strains were grown in RPMI 1640 (Sigma, Italy) at 30 °C for 24 h.

The minimum inhibitory concentrations (MIC), the minimum bactericidal concentrations (MBC), and the minimum fungicidal concentrations (MFC) of the extract, were determined using a broth microdilution, in accordance with the Clinical and Laboratory Standards Institute [31,32]. MIC values were defined as the lowest feijoa EO concentrations showing no bacterial/fungal growth after incubation. The MBC and MFC were defined as the lowest feijoa EO concentration, which killed 99.9% after 24 h incubation at 37 °C, and 24–48 h incubation at 30 °C, respectively. All assays were performed in triplicate.

### 2.8. Statistical Analysis

The results were expressed as the average ± standard deviation (S.D.) of three independent experiments (*n* = 3). A one-way analysis of variance (ANOVA), followed by Bonferroni’s post-hoc comparisons tests, using a SigmaPlot 12.0 and GraphPad prism 5.0 software, were carried out. Statistical significance was considered at *p* < 0.05.

## 3. Results and Discussion

### 3.1. Micromorphological Features

**Figure 1:**
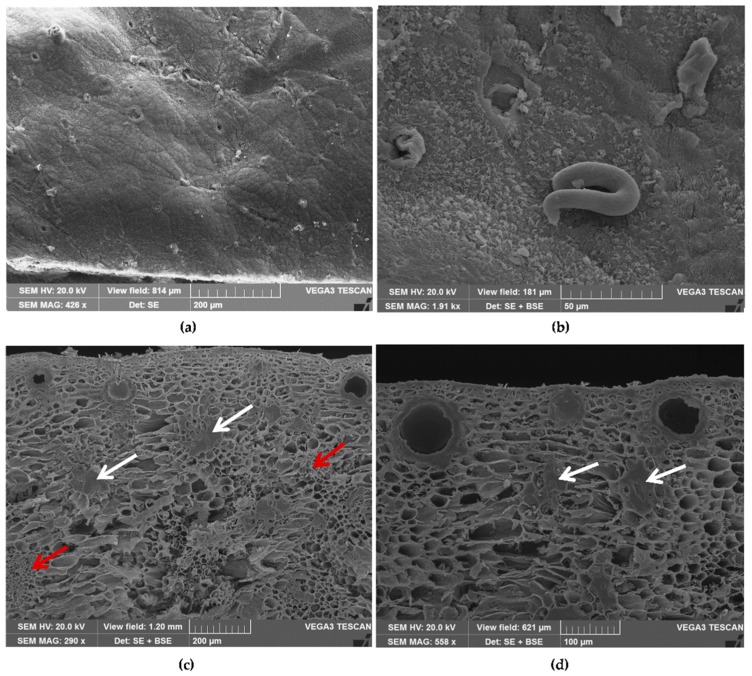
SEM micrographs (**a**–**b**). Epidermis of the exocarp with scattered stomata, openings of secretory cavities, and simple unicellular trichomes (**a**,**b**). Transversal sections of the fruit peel, showing a parenchima rich in schizogenous secretory cavities, located near the epidermis (**c**,**d**), stone cells (**c**,**d**, white arrows), and vascular bundles (**c**, red arrows).

The pericarp of mature fruits is differentiated into three regions: The exocarp, mesocarp, and endocarp. The exocarp is composed of an outer epidermis and a hypodermis. A waxy cuticle covers the epidermis, presenting on its surface a scattered stomata, the openings of secretory cavities, and simple unicellular trichomes (Figure 1a,b). Transversal sections show an epidermal layer and a mesocarp, made up of many layers of parenchyma.

**Figure 2:**
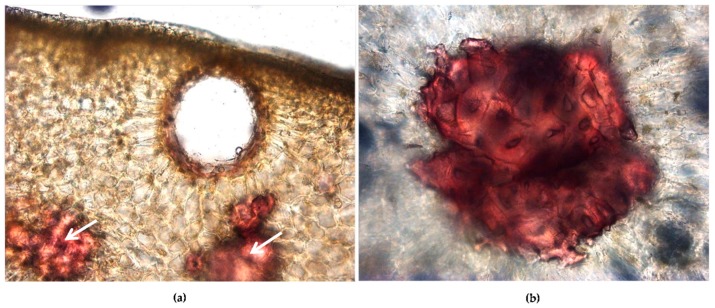
Light microscopy (LM) micrographs (**a**,**b**). Transversal sections of the fruit peel showing a schizogenous secretory cavity, located near the epidermis, (**a**) and many stone cells stained in red (**a**, white arrows and **b**, at higher magnification).

The parenchima is rich in schizogenous secretory cavities, the site of accumulation of essential oils, located near the epidermis and showing different stages of development (Figure 1c,d; Figure 2a); many stone cells are also visible (Figure 1c,d, white arrows; Figure 2a white arrows, and Figure 2b, particularly at higher magnification of red stained stone cells), and several vascular bundles can be found (Figure 1c, red arrows) [33].

### 3.2. Phytochemical Profile of Feijoa EO

The yield in EO obtained was 0.8% (*v/w*), consistently higher than previously reported (< 0.1%) [34]. The amount and quality of feijoa EO could be dependent of the fruit variety, as well as its origin, genotype, soil type, climate, and isolation method [35]. Moreover, an increase in the isolation efficiency was observed when it was carried out immediately after harvesting. Indeed, in this condition, it is possible to avoid peroxidation, isomerization, and rearrangement products, due to light, temperature, and oxygen [36]. The EO composition, with retention index and percentages for each compound, are given in Table 1. 

Forty compounds were identified and quantified. Sesquiterpenes were the most abundant ones (76.89%), followed by monoterpene hydrocarbons (3.26%) and oxygenated monoterpenes (0.34%). The major components of EO were γ-Selinene (17.39%), α-Caryophillene (16.74%), β-Caryophillene (10.37%), and Germacrene D (5.32%). Feijoa EO’s chemical composition depends on the variety, the portion of fruits used for extraction, and the geographical origin. Shaw et al. [34] isolated the EO from the epicarp of a New Zealand feijoa fruit, and obtained an extraction yield < 0.1%. Moreover, the major components identified were, (Z)-Hex-3-en-1-ol (20%), Linalol (18%), Methyl benzoate (14.5%), Germacrene D (6%), and Ottan-3-one (6%) [37,38]. Another study, carried out in France on EO of fruits epicarp, showed a yield comparable with the previous one (< 0.1%), but a completely different chemical constituent profile. Indeed, major components belonged to sesquiterpene group: β-Caryophyllene (12%), Ledene (9.5%), α-Humulene (6.5%), β-Elemene (5%), d-Cadinene (5%), and Ciclo-germacrene (4.5%).

This chemical composition is similar to that obtained by Elfarnini et al. [35], in which sesquiterpenes represent the predominant group of terpenes in feijoa EO. This chemical similarity is probably due to the introduction of the same variety in France and North Africa. Furthermore, a superimposable volatile fraction profile, between feijoa fruits harvested in Georgia (Russia), New Zealand, and Japan, has already been described [39,40]. In light of these pedo-climatic differences, the common features likely arise from the presence of the same variety of feijoas in different geographic areas; indeed, despite a relative abundance variation, the phytochemical profile was preserved. 

In light of this, the phytochemical profile of the feijoa EO may differ, mainly in relation to variety and maturation of plants [35]. In the present study, lower concentrations of 3-ottanone (4.27%), methyl-benzoate (4.46%), and ethyl-benzoate (0.92%) were observed with respect to the previous ones [34,37,41]. Nevertheless, the results are similar to those reported by Elfarnini et al. [35]. Difference observed among studies are ascribed, probably to the fruit variety, and specifically ascribed to the maturation grade. Indeed, the higher amount of Ethyl-benzoate in EO’s volatile fraction is closely related to a more advanced fruits maturity, whereas methyl-benzoate is closely linked to a sweet and perfumed smell, which resembles a strawberry, guava, and pineapple combination. 

### 3.3. Determination of Antioxidant Properties

**Figure 3:**
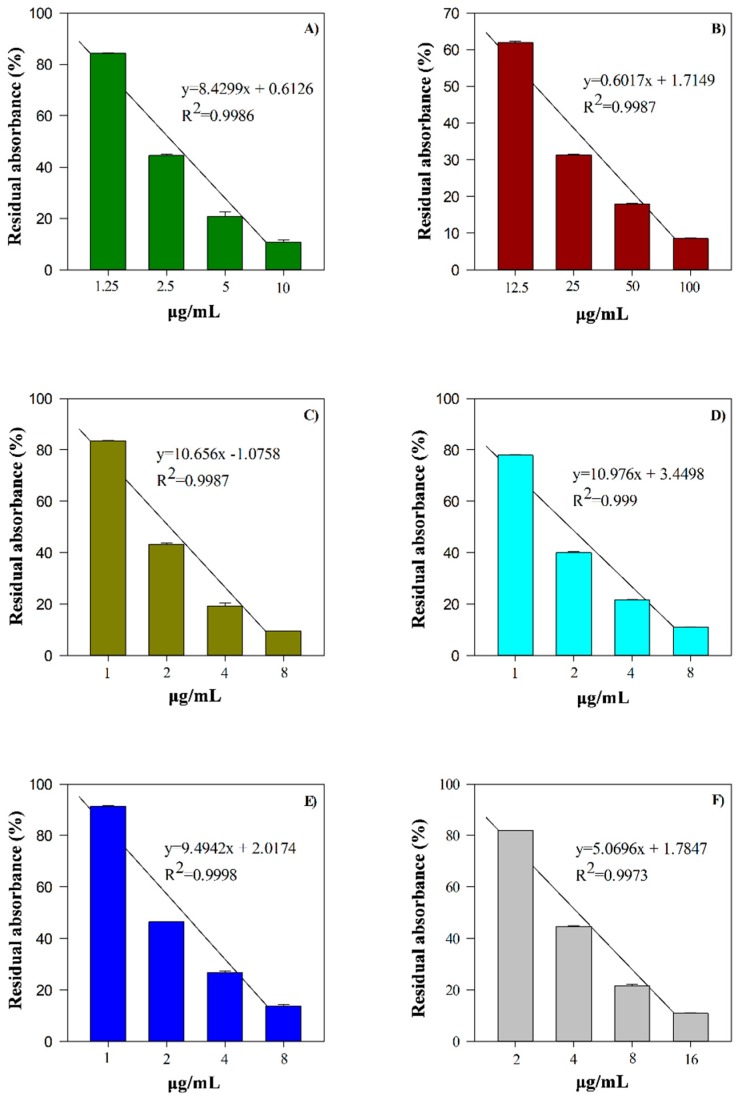
Antioxidant and free radical-scavenging activity of feijoa essential oil (EO) towards Folin (**A**); 2,2-diphenyl-1-picrylhydrazyl (DPPH) (**B**); ferric reducing antioxidant power (FRAP) (**C**); trolox equivalent antioxidant capacity (TEAC) (**D**); iron-chelating activity (**E**), and oxygen radical absorbance capacity (ORAC) (**F**) assay. The asterisks (**) indicate significant differences (*p* < 0.001).

**Figure 4:**
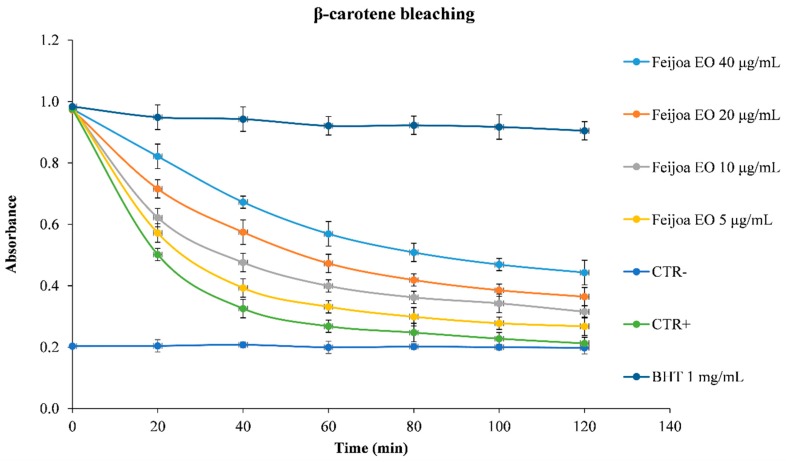
β-Carotene bleaching kinetic curves of the different concentrations of feijoa EO compared to the reference standard butylated hydroxytoluene (BHT) 1 mg/mL.

Oxygen reactive species are related to many diseases, and in light of this, antioxidants play a pivotal role in preserving human health. They can exert their beneficial effects on living organisms by restoring the physiological oxidative balance, as well as modulating biological pathways and membrane function [42]. The determination of the antioxidant and free radical scavenging activity of the EO under study, reported in Figure 3, was carried out using several in vitro cell-free assays, based on different mechanism and reaction environments. Moreover, Figure 4 shows the kinetic curves of different concentrations of the EO, compared to the reference standard BHT. 

Feijoa EO showed a strong dose-dependent antioxidant and free radical scavenging activity in all assays carried out, with the following order of potency expressed as IC_50_: TEAC (3.11 μg/mL, 1.142–8.448) > FRAP (3.87 μg/mL, 2.232–6.723) > Iron-chelating (4.21 μg/mL, 3.551–4.980) > Folin-ciocalteu (4.66 μg/mL, 2.599–8.353) > ORAC (7.65 μg/mL, 6.549–8.939) > DPPH (77.58 μg/mL, 63.377–94.967) (Figure 3).

Feijoa EO, being a source of antioxidant molecules, has the ability to counteract several reactive species, hindering the formation of harmful species, such as hydrogen peroxide, hydroxyl radical, peroxynitrite, and so on. In light of this, it is particularly useful in detoxification mechanisms. In addition, the feijoa EO showed a good iron-chelating activity, which make it potentially useful in reducing the availability of transition metals, lowering the Fenton-like oxidative chain reactions in biological systems, and preserving the integrity and functionality of membranes. Moreover, as highlighted by β-carotene bleaching test results (Figure 4), feijoa EO is able to form adducts with peroxyl radicals. 

Therefore, feijoa EO, not only exhibits strong antioxidant activity, but it is also able to prevent oxidative damage mediated by free radicals in a dose-dependent manner (Figure 3 and Figure 4). These activities can be ascribed predominantly to the EO richness in sesquiterpenes. In particular, the oxygenated sesquiterpenes, which in terms of antioxidant activity, show a similar behavior of the oxygenated monoterpenes, are also present in feijoa EO [43].

### 3.4. Analysis of Cytotoxicity and Cytoprotective Activities

The preliminary test, which was performed for the evaluation of potential cytotoxicity effects of the feijoa EO, did not reveal significant changes in the viability of isolated lymphocytes (monitored by trypan blue coloration) or in hemolysis on isolated erythrocytes versus the control sample in the concentration range 0–40 μg/mL (data not shown). The citoxicity assay, performed by treating lymphocytes with different final amounts of feijoa EO (1.25–150 μg/mL) revealed positive tolerability, of up to 40 μg/mL. On the contrary, there was a net increase in LDH release, a marker of cell membrane integrity, and as a clear consequences, of cell mortality, reaching ~50% at 100 μg/mL, after 24 hours of incubation at 37 °C (data not shown). The detection of erythrocyte haemolysis, due to the presence of feijoia EO in the medium, followed the same behavior as in the case of lymphocytes viability, with a remarkable increase of hemoglobin release in the presence of feijoia EO, higher than 40 μg/mL (data not shown).

**Figure 5:**
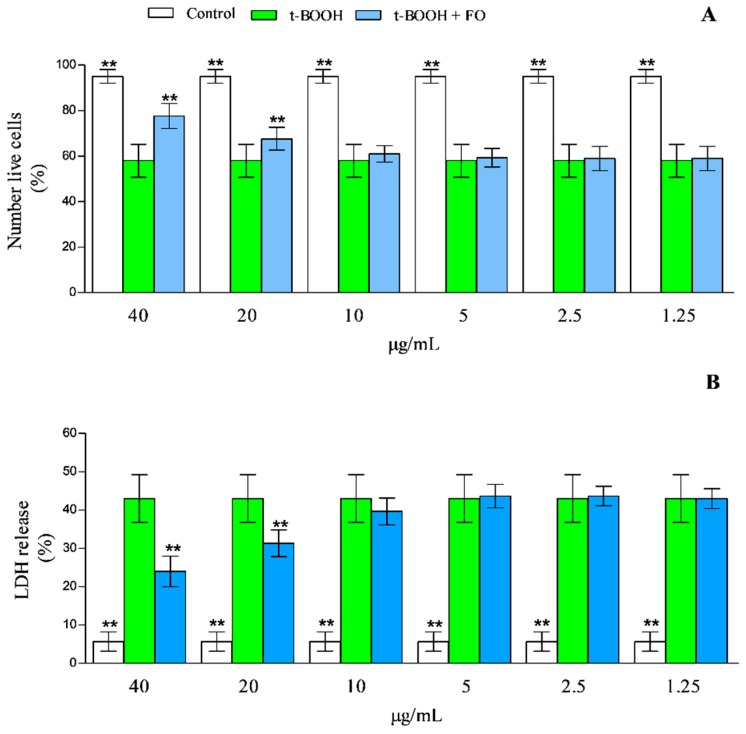
Cytoprotective effects of feijoia EO on t-BOOH treated lymphocytes. Lymphocytes plus 100 μM of t-BOOH have been incubated for 24 h in the absence or in the presence of 100, 75, 50, 25, and 10 μg/mL of feijoia EO. Cell viability and integrity have been analyzed by trypan blue staining (**A**) and LDH release (**B**). The presence of asterisks (**) is indicative of significant differences (*p* < 0.05) of the sample vs t-BOOH-treated one. Each value represents mean ± SD of three independent experiments (*n* = 3).

To test the cyto-protective potential of the feijoia EO up to 40 μg/mL, isolated lymphocytes have been joined with 100 μM of t-BOOH in the absence, or in the presence (1.25–40 μg/mL), of the EO. The results of the experiments, after 24 hours of incubation, are reported in Figure 5. The sample treated with only t-BOOH showed a net increase of cell mortality (~42% fall in cell viability versus the control). The presence of 40, 20, and 10 μg/mL of feijoia EO, resulted in a cell mortality decrease of ~52, 23 to 6%, respectively (Figure 5). At other concentrations, feijoia EO showed effects almost completely negligible, with the results superimposable to those obtained with lymphocytes treated with only t-BOOH. 

The effects of the feijoa EO has been further analyzed by LDH release. In the samples treated with t-BOOH for 24 h a net increase of LDH release was found, resulting in values ~7.2-fold higher than the control (Figure 5). This data decreased of ~45, 21 and 6% in the presence 40, 20 and 10 μg/mL of feijoia EO, respectively. No significant decrease of LDH release has been observed in concentrations below 10 μg/mL.

**Figure 6:**
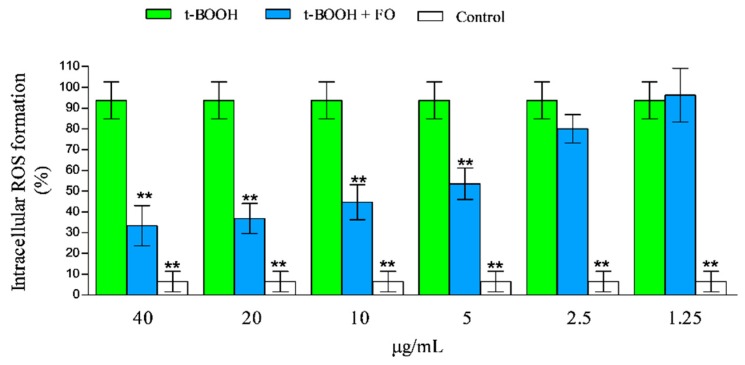
Effects of feijoia EO on t-BOOH-induced reactive oxygen species (ROS) production in erythrocytes. Intra-cellular ROS production was determined by DCF fluorescence. The asterisks (**) indicate significant differences (*p* < 0.05) of the sample vs t-BOOH-treated one. Each value represents mean ± SD of three independent experiments (*n* = 3).

We also investigated the beneficial effects of feijoia EO against t-BOOH-induced alterations of redox status in erythrocytes, measuring the ROS levels. The t-BOOH exposure induced a significant increase in ROS production in erythrocytes, compared to the untreated control (Figure 6). 

The exposure to 40, 20, 10, and 5 μg/mL of feijoia EO was able to reduce t-BOOH-increased ROS production within the cells up to ~ 2.8, 2.5, 2.0, and 1.77-fold, compared with the t-BOOH-treated cells (Figure 6). Basal ROS levels were unaffected by the exposure to the utilized concentrations of feijoia EO (data not shown).

The strong antioxidant activity of the feijoa fruit peel was already described on erythrocytes. Tortora et al. [44] have recently observed that two polyphenol extracts, obtained from feijoa fruit peel and pulp, are protective against mercury-induced toxicity and oxidative stress, with the first one showing the best activity on intact human red blood cells treated with HgCl_2_ [44].

### 3.5. Antimicrobial Properties

The MIC, MBC, and MFC values of the extract are shown in Table 2. Feijoa EO was effective against the Gram-positive bacterial strains of *S. aureus* and *S. epidermidis,* and the yeast *C. albicans*, whereas no effect was observed against the Gram-negative bacterial strains, and the yeasts *C. glabrata* and *C. parapsilosis*. Amongst the Gram-positive bacteria, the ATCC strains of *S. aureus* were more sensitive, compared with *S. epidermidis*, with no significant differences between the ATCC 6538P and the methicillin resistant ATCC 43300. The clinical *S. aureus* strains 14 and 530, isolated from duodenal ulcera, and pharynges, respectively, were the most sensitive, with a bactericidal effect against strain 14 at the concentration of 5.35 mg/mL. 

Amongst the yeasts, *C. albicans* ATCC 10531 and two clinical strains of *C. albicans* (13 and 16) were shown to be the most sensitive, whereas no bactericidal effects were detected against any of the tested yeasts. We recently demonstrated that an EO, extracted from pistachio (*Pistacia vera* L.) hulls, was active against the same Candida species used in the present work [30]. It is well-known that the dramatic rise in antimicrobial resistance poses a major health threat. Antibiotic, antiviral, antifungal and antimalarial resistant infections have increased deaths worldwide. Therefore, novel and natural products are being explored as sources for drug development. Our previous studies have demonstrated that polyphenol-rich extracts, from pistachios (natural raw shelled and roasted salted pistachios) and white grape juice, were effective against a range of bacterial strains, though not effective against yeasts [45,46]. Therefore, the use of feijoa EO, alone or in combination with synthetic drugs, could be proposed as topical agents against both bacterial and fungal skin infections.

## 4. Conclusions

This is the first study which investigates the phytochemical profile, as well as the different biological activities of an EO, obtained from the fruit peel of feijoa var. coolidge. This tasty and functional fruit, less known than other exotic fruits, such as mango and papaya, has recently become more popular in the Italian market. In particular, the tested EO was obtained from fruits harvested in Lametino area (North of Calabria, Italy). 

GC-FID and GC-MS analysis lead to the identification of forty compounds, mainly belonged to sesquiterpenes, monoterpene hydrocarbons, and oxygenated monoterpenes. The most abundant compounds were γ-Selinene (17.39%), α-Caryophillene (16.74%), β-Caryophillene (10.37%), and Germacrene D (5.32%).

Feijoa EO showed a strong and dose-dependent antioxidant activity, which was corroborated by the cytoprotective results, that were observed on lymphocytes pre-treated with t-BOOH, as well as on erythrocytes. These markedly decreased the t-BOOH-induced alterations of the redox status. The antimicrobial screening showed that feijoa EO possesses a selective antibacterial and anti-fungal activity towards the Gram-positive bacteria *S. aureus,* and *S. epidermidis,* as well as the fungi *C. albicans*, respectively. 

These promising biological activities could not be ascribed exclusively to its major components. It has been demonstrated, indeed, that often EOs show a much stronger biological activity than the mainly present compounds, due to their synergic and antagonistic effects, which characterize the plant complexes [21,30,46].

In conclusion, the feijoa EO is an important source of natural antioxidants, with cytoprotective and antimicrobial properties, which are useful in various food, nutraceutical, and pharmaceutical fields. 

In light of this, further investigations are need to validate the potential use of this EO alone, or in combination with synthetic drugs, as topical agents against both bacterial and fungal skin infections.

## Figures and Tables

**Table 1:** Phytochemical profile of *Acca sellowiana* (Berg) Burret fruit essential oil.

#	Compound	Area^1^ (%)	KI^2^
1	3-Octanone	4.27	980
2	3-Octanol	0.20	988
3	Trans-β-ocimene	2.70	1032
4	Cis-β-ocimene	0.56	1043
5	Methyl benzoate	4.46	1084
6	Isomenthone	4.46	1141
7	Ethyl benzoate	0.92	1160
8	2-Undecanone	2.87	1281
9	Methyl geranate	0.28	1309
10	α-Cubebene	1.54	1327
11	Copaene	0.24	1350
12	β-Bourbonene	1.05	1357
13	α-Gurjunene	1.51	1381
14	α-Caryophyllene	16.74	1389
15	β-Cubebene	0.02	1399
16	l-Alloaromadendrene	0.21	1407
17	β-Selinene	0.02	1409
18	β-Caryophyllene	10.37	1421
19	Aromadendrene	0.84	1428
20	2-Isopropil-4 a, 8-dimetil-1,2,3,4,4a,5,6,7-octaidronaftalene	0.24	1434
21	Germacrene D	5.32	1446
22	α-Muurolene	0.19	1453
23	γ-Selinene	17.39	1464
24	β-Gurjunene	2.81	1471
25	2-Tridecanone	0.66	1477
26	γ-Cadinene	0.37	1482
27	l-Calamenene	0.78	1492
28	Naphthalene 1,23,4,6,8a-esaidro-1-isopropil-4,7-dimethyl	0.19	1499
29	α-Calacorene	0.22	1508
30	Selin-4,7(11)-diene	5.12	1529
31	γ-Gurjunene	3.78	1554
32	δ-Guaiene	3.49	1584
33	β-Maaliene	0.14	1593
34	Tau-muurolol	2.83	1605
35	(+)-Ciclosativene	0.10	1611
36	(−)-Isoaromadendrene-(V)	1.58	1614
37	8-isopropil-5-metil-2-metilene-1,2,3,4,4a,5,6,7, octaidro naphtalene	5.16	1617
38	Valencene	0.19	1639
39	Benzyl benzoate	0.31	1722
40	Dibenzoylmethane	0.25	2004
Sesquiterpenes		76.86	
Monoterpenes		3.26	
Oxigenated monoterpenes		0.34	
Others		19.54	

#: Components are listed in their elution order from HP-5-MS column; 1: Values (relative peak area percentage) represent averages of three determinations (*n* = 3); 2: Retention index (KI) relative to standard mixture of n-alkanes on HP-5MS column.

**Table 2:** Minimum inhibitory concentrations (MIC), minimum bactericidal concentrations (MBC), and minimum fungicidal concentrations (MFC) values (mg/mL) of feijoa essential oil against bacterial and fungal species.

**Strain**	**Feijoa EO**	
	**MIC**	**MBC**
*S. epidermidis* ATCC 35984	5.35	> 5.35
*P. aeruginosa* ATCC 9027	> 5.35	> 5.35
*E. coli* ATCC 10536	> 5.35	> 5.35
*S. aureus* ATCC 43300	2.67	5.35
*S. aureus* ATCC 6538P	2.67	5.35
*S. aureus* strain 8	> 5.35	> 5.35
*S. aureus* strain 6	5.35	> 5.35
*S. aureus* strain 530	2.67	> 5.35
*S. aureus* strain 14	2.67	5.35
*S. aureus* strain 808	5.35	> 5.35
*S. aureus* strain 526	5.35	> 5.35
*S. aureus* strain 84	5.35	> 5.35
**Strain**	**MIC**	**MFC**
*C. albicans* ATCC 10531	2.67	> 5.35
*C. albicans* strain 12	5.35	> 5.35
*C. albicans* strain 13	2.67	> 5.35
*C. albicans* strain 16	2.67	> 5.35
*C. glabrata* strain 9	5.35	>5.35
*C. glabrata* strain 33	5.35	> 5.35
*C. parapsilosis* strain 30	5.35	> 5.35
*C. parapsilosis* strain 34	5.35	> 5.35
